# Reproductive performance, inbreeding evolution, and genetic diversity of the Venezuelan Carora cattle evaluated through pedigree analysis

**DOI:** 10.3389/fvets.2025.1696434

**Published:** 2025-12-17

**Authors:** Luis F. Cartuche-Macas, José R. Perez-Gonzalez, Ernesto J. Jimenez-Quintero, Miguel A. Gutiérrez-Reinoso, Joar Garcia-Flores, Manuel García-Herreros

**Affiliations:** 1 Escuela Superior Politécnica Agropecuaria de Manabí Manuel Félix López (ESPAM), Carrera de Medicina Veterinaria, Calceta, Ecuador; 2 Universidad Politécnica Territorial de Maracaibo (UPTM), Maracaibo, Venezuela; 3 Asociación de Criadores de Ganado Carora (ASOCRICA), Barinas, Venezuela; 4 Laboratorio de Biotecnología Animal, Facultad de Ciencias Veterinarias, Departamento de Ciencia Animal, Universidad de Concepción (UdeC), Chillán, Chile; 5 Facultad de Ciencias Veterinarias, Universidad UTE, Quito, Ecuador; 6 Instituto Nacional de Investigação Agrária e Veterinária (INIAV), Santarém, Portugal; 7 CIISA-AL4AnimalS, Faculty of Veterinary Medicine, University of Lisbon, Lisbon, Portugal

**Keywords:** reproduction, inbreeding, genetic variability, diversity loss, Carora breed, dairy cattle

## Abstract

The native Carora dairy breed was created in Venezuela with the objective of improving cattle adaptation to tropical conditions and increasing the production performance. The aim of this research was to study the reproductive performance, inbreeding evolution, and genetic diversity of the indigenous Carora breed using official pedigree information from the Carora Cattle Association of Venezuela. The total population included 72,411 females and 8,067 males. Six databases were considered: historical (all individuals born between 1985 and 2024 = 80,473), four populations born from 1985 to 2024 taken at 10-year intervals (1985–1994; 1995–2004; 2005–2014; 2015–2024) that included 8,442, 15,694, 34,987, and 21,350 individuals, and reference population (individuals with known sire and dam within of the population in the last period). Population structure variables were pedigree completeness index (PCI); number of equivalent (GEq), complete (GCom); and maximum (GMax) generations; and generation interval (GI). GD variables were inbreeding (F), inbreeding increment (ΔF), average relatedness (AR), co-ancestry (C), non-random mating (*α*), effective population size (Ne), and genetic conservation index (GCI). The gene origin probability variables were number of founders (f), effective number of founders (fe) and ancestors (fa), number of equivalent genomes (fg), fe/fa and fg/fa ratio, and GD losses. The databases were analyzed by ENDOG, POPREP, CFC, Re-laX2, and GRAIN v.2.2 software. PCI in the historical population compared to the population of the last period increased from 60.54 to 70.93% in the first generation, while the GI decreased from 5.78 to 5.95 years in the historical and last period, respectively. Moreover, *F* = 2.35%, AR = 4.04%, ΔF = 0.43%, C = 2.02%, *α* = 0.0034; GCI = 2.27, and Ne-Coan = 132.53 values were obtained in the last period. Gene origin probability in the reference population was fa = 28, fe = 58.69, fg = 27.74, fg/fe = 2.10, showing a GD loss due to unequal contribution of founders (0.85%) and bottleneck and genetic drift (0.95%). In conclusion, the native Carora cattle population displayed low-average genetic diversity, and its inbreeding value increased over time. Thus, conservation strategies, such as introducing new purebred bloodlines, establishing gene banks, and developing genetic improvement programs, would be crucial to minimize the future inbreeding to prevent the GD loss in native Carora cattle.

## 1 Introduction

The Carora cattle breed was developed around a century ago in central-western Venezuela, crossbreeding the native Amarillo de Quebrada Arriba cattle and the exotic Brown Swiss breed. The former breed was used to fix genes adapted to tropical conditions, and the latter to increase milk production (1). In 1979, the Carora Breeders Association (ASOCRICA) was formed and officially recognised in 1982. From this point onwards, genealogical records of founding herds began to be managed, and in 1992, it was opened up to the general public through a cattle cross-breeding programme, defining the Carora breed as those individuals containing ≥87.5% Carora/Brown Swiss purity and at least 12.5% Carora founders (2). Thus, adaptive traits of interest were enhanced, such as heat tolerance given by the SLICK gene (3, 4), efficient use of low-nutritional-quality feed when grazing (5), hardiness and adaptation to adverse climatic conditions (6), high milk production in tropical conditions (7), and high fertility and longevity, reaching one birth per year on a continuous basis (1).

Within genetic improvement programmes, one of the main objectives was to reduce the loss of genetic diversity by monitoring inbreeding and its associated parameters, such as average relatedness (AR), inbreeding coefficient (F), effective size population (Ne), among others, using genealogical and molecular information (8, 9) in order to establish strategies to prevent the appearance of deleterious genes and inbreeding depression (10, 11). Regarding this issue, several studies have been conducted on the Carora breed. For example, at the genealogical level, Tullo (12) showed that this breed has not been in danger of extinction, as it maintained low levels of inbreeding between 1983 and 2010. Moreover, genetic diversity at the molecular level has been shown to be highly variable, with five population subtypes observed (13), as well as high variability in milk proteins (14). Furthermore, inbreeding has traditionally been estimated using the classic method of Wright (15), with the development of more efficient algorithms in both estimation and genealogy processing times. These include the algorithms of Meuwissen and Luo (16) and Sargolzaei et al. (17), implemented in computer programs such as ENDOG (18), CFC (17), RelaX2 (19), EVA (20), optiSel (21), among others. Given the great variability of population structures at the genealogical level in terms of ancestor information, alternative methodologies have been designed, such as VanRaden’s recursive algorithm (22), which considers that animals with unknown parents cannot necessarily be considered as such, but rather that they have the average inbreeding coefficient of animals with known parents born in the same year. This methodology was implemented in the BLUPF90 programme developed by Aguilar and Misztal (23).

Thus, the main objective of the present study was to determine the inbreeding evolution, reproductive performance, and genetic diversity of the native Venezuelan Carora cattle using official genealogical information in order to determine the population evolution and current status of this breed regarding genetic diversity loss, as well as to identify the potential factors that influence its breeding and genetic selection.

## 2 Materials and methods

### 2.1 Ethical statement

The study was conducted according to the guidelines of the Declaration of Helsinki and by following the Code of Ethics for animal experiments as reflected in the ARRIVE guidelines available at http://www.nc3rs.org.uk/ARRIVEchecklist (Accessed on 2 January 2025). However, the present research did not require any ethical review and approval by a specific Bioethics Committee for the use of experimental animals since the study was directly carried out using the records and databases provided by Carora Breed Cattle Association (ASOCRICA, Venezuela). Then, the studies were conducted in accordance with the local legislation and institutional requirements.

### 2.2 Genealogical database

The genealogical database was provided by the Carora Creole Cattle Association (ASOCRICA). A total of 80,478 registered animals were used, including 8,067 bulls and 72,411 cows, born between January 1985 and December 2024. For the analysis, several populations were considered: (i) historical population (all individuals born between 1985 and 2024); (ii) four populations born from 1985 to 2024 taken at 10-year intervals (1985–1994; 1995–2004; 2005–2014; and 2015–2024) that included 8,444, 15,694, 34,987, and 21,350 individuals, respectively; and (iii) reference population (encompassing individuals with known sire and dam from the populations described above). Populations were defined because calculations related to genetic diversity, gene origin probabilities, and founder analyses can only be performed by considering only individuals with both parents known or by comparing them with historical and current data sets as suggested by Navas et al. (24). ENDOG (v 4.8) software (18) and POPREP (25) were used to perform demographic and genetic analyses to quantify and trace genetic diversity back to ancestors.

### 2.3 Demographic structure

The pedigree completeness index (PCI) was calculated following the assumptions of Navas et al. (24) from the first to the fifth generation, and also the number of maximum generations (GMax), number of complete generations (GCom), and number of equivalent generations (GEqu) in the five defined populations.

The generation interval (GI) was calculated for the four gametic pathways from sire and dam to son and daughter, respectively, according to James (26). For this purpose, the record of the birth date of each individual, together with that of its parents, was used.

### 2.4 Inbreeding and coancestry

Inbreeding coefficient (F): The F has been defined as the probability that two alleles taken at random are identical per offspring. The F was estimated using the algorithm proposed by Meuwissen and Luo (16), and the increase in inbreeding (ΔF) per generation was calculated using the equation proposed by Gutiérrez and Goyache (18):


ΔF=Ft−Ft−11−Ft−1

where Ft and Ft-1: average inbreeding of the n^th^ generation (i = 1,…, t).

Additionally, the inbreeding coefficient was estimated using the recursive method based on the algorithm proposed by Aguilar and Misztal (23), which assumes that the unknown parents do not have an inbreeding coefficient of zero, but assumes that it is equivalent to the average inbreeding coefficient of parents born in the same year.

Average relatedness (AR): Each individual’s average relatedness coefficient was defined as the probability that two related individuals have inherited a particular allele of a single locus/gene from their common ancestor (this allele is known as IBD: identical by descent). AR was defined as the probability that a randomly selected allele from a population belongs to a specific individual, which was calculated using the vector c, where each element corresponds to the respective AR of an individual, defined by Gutierrez et al. (27).

Coancestry (C): The C between two individuals is the probability that the genes, taken at random from each of the individuals, are identical by descent (28). As a result, the C between two individuals is the F of their potential offspring. The C between two individuals is equal to the inbreeding coefficient of their offspring if the individuals are related (29). It was also used to analyse the degree of relatedness and non-random mating, *α* within breeds. The coancestry was estimated according to the algorithm of Colleau and Sargolzaei (30).

Non-random mating (α): The α was estimated as the correlation of genes between two individuals in relation to the correlation of genes taken at random from the population (α) according to Caballero and Toro (31). It indicates the degree of deviation from Hardy–Weinberg proportions and is related to the inbreeding coefficients according to Wright (32):


$$\left(1-F\right)=\left(1-C\right)\left(1-\alpha \right)$$

Genetic Conservation Index (GCI): The GCI was estimated from the genetic contribution of all founders, considering the proportion of genes from a founder animal in the pedigree under analysis according to Wang, Zhou (33). The following equation was used:


GCI=1∑pi2

where “pi” is the proportion of genes of founder “i” in the individual’s pedigree.

### 2.5 Effective population size (Ne)

The Ne method used to compute the effective population size was based on the following equation:


$$Ne=\frac{1}{2\times \Delta F}$$

where ΔF is used according to the Ne-estimated method, such as equivalents complete generations (Ecg), coancestry (Coan), and generation interval (Ln).

Ne-Ecg was based on the following equation:


$$\Delta F_{i}=1-\sqrt[{Ecg-1}]{1-F_{i}}$$

where Ecg = sum of all known ancestors with ${\left(\frac{1}{2}\right)}^{n}$ ; Fi = individual inbreeding coefficient.

Ne-Ln was based on the following formula:


$$\Delta F_{\text{ln}}=\left(-1\right)bL$$

where b = slope from the logarithmic regression of ln (1 − F) on year of birth; L = generation interval.

Ne-Coan was based on the following equation:


$$\Delta F_{g}=\frac{f_{t}-f_{t-1}}{1-f_{t-1}}$$

where f_t_ = additive genetic relationship (AGR) of offspring; f_t − 1_ = AGR of parents.

The methods were implemented in POPREP software (27, 29).

### 2.6 Gene origin probability and ancestral contributions

Number of founders (f): The term “f” is defined as those individuals with unknown parents, assumed to be unrelated, and have an inbreeding coefficient of 0.

Effective number of founders (fe): The term “fe” is defined as the number of founders that contribute equally and are expected to produce the same genetic diversity as the study population. It was estimated from the following equation, Lacy (34):


$$f_{e}=\frac{1}{\sum_{k=1}^{f}q_{k}^{2}}$$

where “qk” is the gene origin probability from ancestor “k,” and “f” is the real number of founders.

Effective number of ancestors (fa): The fa was defined as the minimum number of ancestors that are not necessarily founders and that account for the full genetic diversity of a population according to Boichard, Maignel (35):


$$f_{a}=\frac{1}{\sum_{k=1}^{f}p_{k}^{2}}$$

where “pk” is the marginal contribution of an ancestor “k” that is not explained by other chosen ancestors, and “f” is the real number of founders.

Number of founder genome equivalents (fg): The term “fg” is defined as the number of founders that would be expected to produce the same genetic diversity as the population under study if the founders were equally represented and no allele loss occurred. This was estimated from twice the inverse of the average C according to Caballero and Toro (31). The number of ancestors contributing to 25, 75, and 100% of the genetic pool was calculated as those that contributed to 25, 75, and 100% of the genes, respectively (fa25, fa75, and f100). The genetic bottleneck was determined by calculating the number of ancestors in the population that contributed to 50 percent of the genes (fa50). The fa is expected to be smaller than the fe in the presence of a bottleneck, which can be indicated by the fe/fa ratio.

Genetic contributions: The genetic contributions were estimated for the top ten ancestors with the maximum genetic impact between 1999 and 2023. The marginal contribution of each major ancestor “j” was calculated as proposed by Boichard et al. (35). The CFC v.1.0 software was used to calculate genetic ancestral contributions and gene origin probabilities (36).

### 2.7 Genetic diversity

Genetic diversity (GD): The GD was estimated using the equation:


GD=1−12fg

Genetic diversity loss (GD-loss): The population GD-loss from the founder generation was estimated using 1 - GD. The GD-loss due to unequal contribution of founders was estimated according to Caballero and Toro (31) using 1 - GD*:


GD∗=1−12fe

The unequal contribution of founders relates to the fact that the genetic contributions of founders of specific populations may be of different proportions due to past directional mating (human-mediated or not) during the process of population shaping. The difference between GD and GD* indicates the GD-loss due to genetic drift accumulated from the population founding (34) and the effective number of non-founders (Nenf).

### 2.8 Data analysis and software

The software used for the database analysis were ENDOG v. 4.8 (18), POPREP (25), CFC (17), RelaX2 (19), and GRAIN package version 2.2 (37, 38) by means of which the demographic-derived parameters, genetic diversity indices, and gene origin probability were obtained. Moreover, the software RELAX2 and the GRAIN package were applied to calculate recursive inbreeding.

### 2.9 Statistical analysis

All descriptive statistical analyses were carried out using the statistical software package SPSS for Windows® v.26 (IBM Corp., Chicago, IL, USA) using the database containing different parameters. General statistical analysis was performed using one-way ANOVA for the analysis of the number of generations, generation intervals in the four gametic pathways, and chronological periods. Pair-wise comparisons of mean value were conducted by Student’s *t*-test. The level of significance was set at *p* < 0.05.

## 3 Results

### 3.1 Pedigree completeness-derived parameters

Pedigree completeness integrity (PCI) showed maximum values of 70.93% in the first generation of the last period studied, indicating that there has been no improvement in pedigree integrity within the 40 years evaluated (Table 1). Complete pedigree generations showed values between 1.0 and 1.80, indicating incomplete records, considering that despite the maximum and equivalent generations being between 12.37 and 5.28, respectively, in the 2015–2024 period, which suggests that despite incomplete records, the population retains diverse genetic contributions from more distant ancestors.

**Table 1:** Pedigree completeness-derived parameters in Carora cattle breed raised in Venezuela.

Pedigree completeness-derived parameters	Historical	1985–1994	1995–2004	2005–2014	2015–2024
Population of animals with pedigree	80,473	8,442	15,694	34,987	21,350
Number of generations (n)	18	10	12	16	18
1st generation (%)	60.54	63.45	55.63	52.15	70.93
2nd generation (%)	56.71	57.49	52.63	48.91	67.79
3rd generation (%)	52.83	48.88	49.98	46.83	65.62
4th generation (%)	48.79	39.85	46.11	45.14	64.06
5th generation (%)	44.79	32.59	40.81	43.17	62.57
Average GMax	9.51	4.95	7.65	9.71	12.37
Average GCom	1.34	1.00	1.16	1.22	1.80
Average GEqu	3.93	2.23	3.10	3.89	5.28

GMax: Maximum number of generations; GenCom: Complete number of generations; GEqu: Equivalent number of generations; *the historical period included individuals/data before 1985.

### 3.2 Reproductive performance derived-parameters

Table 2 shows the population structure and reproductive performance of the Carora breed during the different periods analysed. A general decrease in the population was observed in the last period, although the number of sires increased and the number of dams decreased slightly. Thus, the number of individuals producing offspring decreased considerably throughout all periods, considering that in the period 2005–2014, the value was 38.36%, and in the period 2015–2024, it was 17.05%, with the opposite effect for individuals without offspring. In addition, it was observed that in the period 2005–2014, there was a significant increase in individuals (11,141, 2,483, and 2,973) with only a known sire, a known dam, and no known parents, respectively.

**Table 2:** Population structure and reproductive performance-derived parameters in Carora cattle raised in Venezuela from 1985 to 2024.

Demographic-derived parameter	Historical	1985–1994	1995–2004	2005–2014	2015–2024
Number of animals with pedigree	80,473	8,442	15,694	34,987	21,350
Number of animals (reference population)	46,748	5,312	8,587	18,390	14,459
Dams (total)	32,100	3,597	5,837	12,318	10,348
Sires (total)	2,679	227	480	932	1,040
Individuals with progeny (offspring)	28,588	4,158	7,368	13,422	3,640
Individuals without progeny (offspring)	51,885	4,280	8,326	21,565	17,710
Number of animals with both known parents	46,748	5,312	8,587	18,390	14,459
Number of animals only with known sire	23,695	2,594	5,424	11,141	4,536
Number of animals only with known dam	5,220	271	1,022	2,483	1,444
Number of animals with no known parents	4,810	265	661	2,973	911

Reproductive-derived parameter	Historical	1985–1994	1995–2004	2005–2014	2015–2024
Total number of females	72,409	7,855	14,125	31,574	18,855
Number of females with offspring	26,614	3,901	6,961	12,507	3,245
Calving’s ratio (%)	36.76	49.66	49.28	39.61	17.21
Number of calves born (offspring)	115,956	19,037	37,469	50,516	8,934
Average number of calves per sire	43.28	83.86	78.06	54.20	8.59
Maximum number of calves per sire	1,098	774	1,098	920	182
Average number of calves per dam	3.61	5.29	6.42	4.10	0.86
Maximum number of calves per dam	25	17	25	24	7

**Figure 1:**
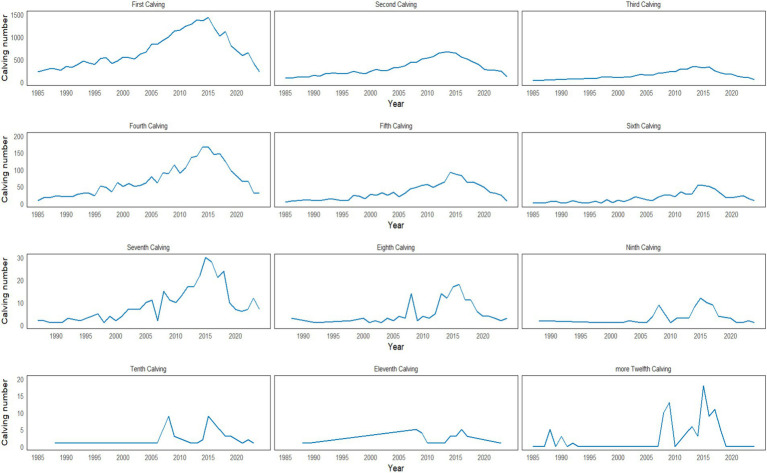
Reproductive performance in Carora creole cattle (births from 1985 to 2024).

The number of females included in the pedigree showed a decrease of approximately 60%, coupled with a 25.95% reduction in cows with offspring in the last period. When analysing the average number of calves per sire and dam, this also diminished from 83.86 to 8.59 and from 5.29 to 0.86 calves, respectively, between the periods 1985–1994 and 2015–2024. Figure 1 shows the evolution of the number of births (1 to 12) per year. It was observed that in Carora breed, there were individuals that reached more than 12 births, although the most frequent were 7–8 births/dam.

### 3.3 Generation intervals

**Figure 2:**
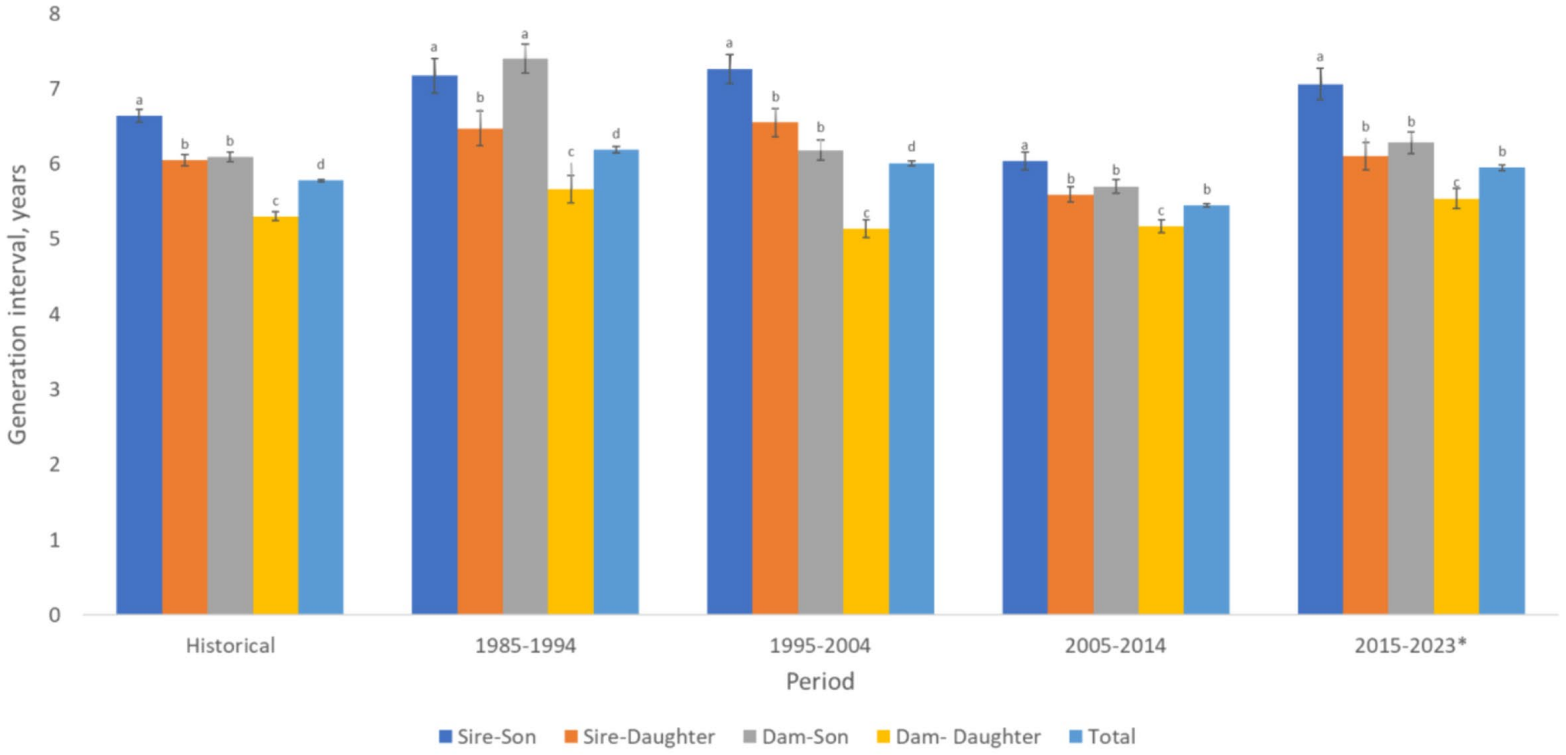
Generation interval in the four gametic pathways and periods in Carora cattle raised in Venezuela. Mean ± S. E. M. Different letters **(A-D)** in bars within the same period show statistical differences among gametic pathways (*p* < 0.05). *Data collected up to the year in which the breeder had his last calving (2023).

The GI for each gametic pathway and period is shown in Figure 2. The total GI showed values between 5.45 and 6.19 years during the periods evaluated, while analysis by gametic pathway showed that the sire-son and dam-son pathways remained between 6 and 7 years, respectively, and the sire-daughter pathway, approximately 6 years. Finally, the dam-daughter gametic pathway showed values approximately 5.5 years throughout the different periods. The period with the lowest generation interval in the four pathways was 2005–2014, while the period 2015–2023 showed a slight increase. The sire-son pathway was significantly different compared to the other pathways, while no differences were observed in the sire-daughter, dam-son, and dam-daughter pathways (Figure 2).

### 3.4 Inbreeding, average relatedness, coancestry, and non-random mating

Table 3 shows the results of inbreeding and its derived parameters for each period evaluated. The mean inbreeding coefficient observed between periods ranged between 0.84 and 2.35%, showing an upward trend except in the 2005–2014 period. Upon analysis, the inbreeding coefficient estimated using the recursive method was found to be 5.88%, with a continuous upward trend.

**Table 3:** Inbreeding (F), average relatedness (AR), coancestry (C), and non-random mating (*α*) in Carora cattle raised in Venezuela from 1985 to 2024.

Parameter	Historical (80,473)	1985–1994 (8,442)	1995–2004 (15,694)	2005–2014 (34,987)	2015–2024 (21,350)
Inbreeding coefficient (F, %)	1.76	0.84	1.69	1.65	2.35
Inbreeding for recursive method (%)	5.19	2.74	4.87	5.50	5.88
Inbreeding increment (ΔF, %)	0.43	0.46	0.55	0.38	0.43
Maximum inbreeding coefficient (%)	37.87	31.25	37.87	37.65	34.52
Inbred animals (%)	50.53	24.72	48.62	49.28	64.18
Highly inbred animals (%)	1.61	1.94	2.19	1.23	1.69
Coancestry coefficient (C, %)	1.83	1.45	1.84	1.80	2.02
Average relatedness (AR, %)	3.66	2.90	3.68	3.60	4.04
Genetic conservation index (GCI)	2.09	2.05	2.09	1.99	2.27
Non-random mating (α)	−0.0007	−0.0062	−0.0015	−0.0015	0.0034

Moreover, in related individuals, the value reached 64.18% in the last period, and the maximum inbreeding coefficients, ranging from 31.25 to 37.84% (Table 3). Coancestry, average kinship, and GCI showed an increasing trend, reaching values of 2.02, 4.04, and 2.27, respectively. Meanwhile, *α* in the last period showed a positive value of 0.0034.

### 3.5 Effective population size (Ne)

**Figure 3:**
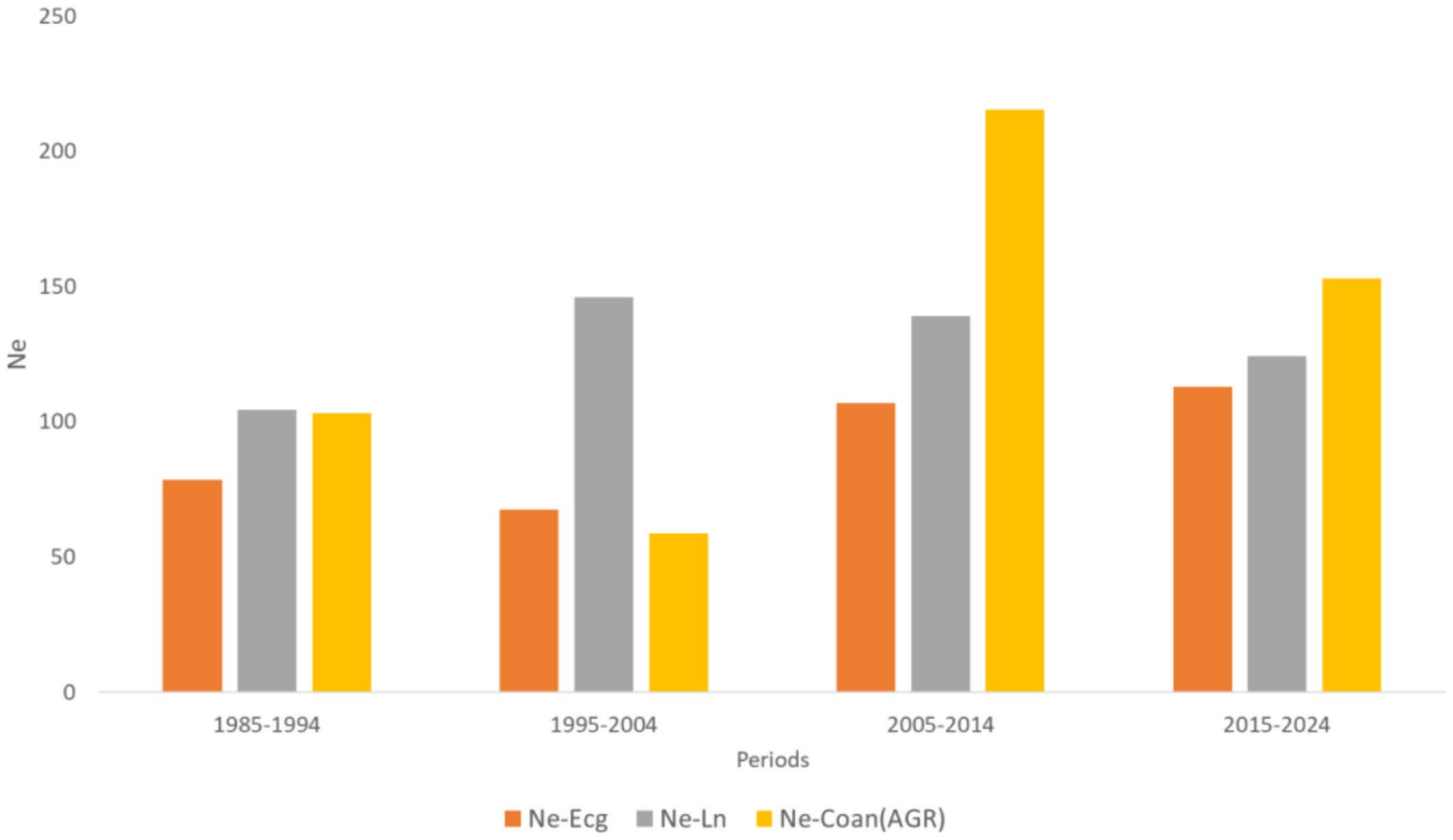
Evolution of the effective size (Ne) in Carora cattle raised in Venezuela from 1985 to 2024. Ne-Ecg: obtained from animals with their complete ancestors born in generation *t*; Ne-Ln: obtained from animals born in generation *t*; Ne-Coan: obtained from animals born in generation *t + 1* and *t*.

The effective population size (Ne) estimated by the Ne-Ecg, Ne-Ln, and Ne-Coan methods was 91.53, 128.43, and 132.53, respectively. All three methods showed a reduction in Ne value in the last period evaluated, with values between 113.00 and 152.80 (Figure 3).

### 3.6 Gene origin probability, ancestral contributions, and genetic diversity

#### 3.6.1 Gene origin probability and ancestral contributions

Table 4 shows the results of the analysis of the gene origin probability. The analysis showed a reduction in the number of founders and ancestors contributing to the reference population, as well as in the effective number of founders, ancestors, and equivalent founder genomes, reaching values of 52.58, 28, and 27.74, respectively. The values of the fe/fa, fg/fe, and fg/fa ratios tend to decrease as well. As for the number of ancestors representing genetic diversity, it was observed that three ancestors represented 25% in all periods, while 50% of genetic diversity was reduced from 12 to 9 ancestors, and 75% of genetic diversity decreased from 40 to 32 ancestors.

**Table 4:** Gene origin probability and ancestral contributions in Carora cattle raised in Venezuela from 1985 to 2024.

Gene-origin/Ancestral contribution parameters	Historical*	1985–1994	1995–2004	2005–2014	2015–2024
Historical population (n)	80,473	8,442	15,694	34,987	21,350
Reference population (n)	46,748	5,312	8,587	18,390	14,459
Base population (one or more unknown parents)	4,810	265	661	2,973	911
Current base population (one unknown parent = half founder)	28,915	2,865	6,446	13,624	5,980
Number of founders contributing to the reference population (n)	10,966	1,792	2,761	6,286	5,489
Number of ancestors contributing to the reference population (n)	10,487	1,658	2,538	5,851	5,024
Effective number of non-founders (Nenf)	127.03	423.46	124.02	85.13	52.58
Effective number of founders (fe)	42.93	82.03	51.21	60.42	58.69
Effective number of ancestors (fa)	28	32	24	28	28
Founder genome equivalents (fg)	32.09	68.72	36.24	35.34	27.74
fe/fa ratio	1.53	2.56	2.13	2.16	2.10
fg/fe ratio	0.75	0.84	0.71	0.58	0.47
fg/fa ratio	1.15	2.15	1.51	1.26	0.99
Number of ancestors to explain:					
25% of gene pool	3	3	3	3	3
50% of gene pool	10	12	8	10	9
75% of gene pool	37	40	31	36	32
100% of gene pool	10,487	1,658	2,538	5,851	5,024

fe/fa ratio: effective number of founders (fe) / effective number of ancestors (fa); fg/fe ratio: founder genomes equivalent (fg) / effective number of founders (fe); fg/fa ratio: founder genomes equivalent (fg) / effective number of ancestors (fa).

#### 3.6.2 Genetic diversity loss

Table 5 shows the genetic diversity loss in each evaluated period. The genetic diversity loss was greater in the period 2015–2024, with the greatest genetic loss caused by genetic drift during the last period, contrary to the three previous periods, which were caused by the unequal contribution of founders.

**Table 5:** Genetic diversity loss in Carora cattle raised in Venezuela from 1985 to 2024.

Genetic diversity parameters	Historical	1985–1994	1995–2004	2005–2014	2015–2024
GD (%)	98.44	99.27	98.62	98.59	98.20
1-GD (GD loss)	1.56	0.73	1.38	1.41	1.80
GD* (%)	98.84	99.39	99.02	99.17	99.15
Proportion of unequal contributions of the founders in GD loss (%)	1.16	0.61	0.98	0.83	0.85
Proportion of random genetic drift in GD loss (%)	0.39	0.12	0.40	0.59	0.95
Proportion of random genetic drift and bottlenecks in GD loss (%)	1.56	0.73	1.38	1.41	1.80

## 4 Discussion

This is the first report on the determination of the inbreeding evolution, reproductive performance, and genetic diversity of the Carora cattle in Venezuela using official genealogical information in order to determine the population evolution and current status of this breed regarding genetic diversity loss. In the present study, the pedigree completeness determined in the first three generations could be considered low compared to other native breeds, such as some Iberian breeds like the Negra Andaluza (32.60–98.46%) and the Berrenda Negra and Colorada (82.76 and 79.57%, respectively) (39, 40), as well as other Latin American Creole breeds such as the Costeño con Cuernos (20.4–100%), Sanmartinero (41.0–97.5%), Blanco Orejinegro (3.6–100%), and Romosinuano (41.3–94.3%) (41, 42). Pedigree completeness is related to the number of equivalent generations, which indicates the average number of complete generations known at the individual level. In the case of the Carora breed, in the last period studied, the number of equivalent generations value was 5.28, greater than those of the Berrenda Negra and Colorada breeds (1.61 and 1.54, respectively) (40) and other Latin American creole breeds described above (41, 42). The trend in the population structure parameters observed in the Carora cattle was similar to that of the Venezuelan Criollo Limonero cattle (43), despite their divergent population sizes. For example, the population size of Criollo Limonero cattle between 2014 and 2023 was 462; however, the population size of Carora cattle in the present study was 21,350.

From a reproductive standpoint, there was a decrease in the average number of offspring per sire from 83.86 to 8.59, which differs from the Criollo Limonero breed, which showed a value of 5.25 to 0.42 (43) and the Negra Andaluza breed, with an average value of 3.87 (39). This fact could indicate a reduction in the age of individuals in artificial insemination centres, an increase in sires available for breeding or collection, and/or a considerable reduction in the number of females available, as shown in the Venezuelan Criollo Limonero cattle described above, with 235 sires and 227 dams (43). Additionally, it was observed that the maximum number of offspring per sire was between 182 and 1,098, probably because ASOCRICA has had an artificial insemination centre since 1968 with a wide range of proven and trial sires, whose semen has been distributed nationwide (44). Moreover, the number of offspring per female when comparing different periods showed values from 5.29 to 0.86, which was greater than the Criollo Limonero cattle, which showed values from 2.69 to 0.43 (43); however, the lowest value from both was considered very low compared to the Negra Andaluza breed, which had values of 1.64 to 1.53 (39). These results could be due to reproductive problems in the animals or to the fact that many of the females were not inseminated properly, as the dams did not produce calves annually.

Regarding the generation interval, the estimated value was 6.19 years, which is high compared to improved cattle breeds, such as European red dairy breeds, which showed values of 4.8 years for animals born between 2010 and 2018 (45), or the Jersey and Holstein Friesian breeds of northern Europe with values between 4.7 and 5.5 years (46). However, when compared to other native breeds, the value was similar, as is the case with the Venezuelan Criollo Limonero cattle with 6.19 years (43), and the Istrian cattle (IC), Slavonian-Syrmian Podolian cattle (SSP), and Busha (BS) breeds from Croatia with values of 6.93, 6.12, and 5.44 years, respectively (47), or also the case of the Colombian breeds such as Romosinuano with 6.36 years (42) and Blanco Orejinegro with 4.58 years (48). Furthermore, the Carora cattle GI value was lower compared to the Brazilian Zebu breeds (Brahman, Gir, Guzerat, Indubrasil, Nelore, Sindi, and Tabapua), which had values between 6.95 and 9.78 years (49). The high GI value in the Carora cattle could be due to the drastic reduction in the population census, similar to that observed in the Indubrasil cattle from Brazil (49).

When analysing the genetic pathways, it was observed that the values were similar between the sire-son/sire-daughter pathway with 6–7 years, which is a high value compared to the improved Holstein Friesian and Montbeliarde dairy breeds in Ecuador, which showed values of 3.33–4.33 years and 3.92–6.37 years, respectively (50, 51); however, the highest GI was observed in the Charolais breed in Ecuador, which was estimated at approximately 10 years (52, 53). When comparing this paternal GI pathway with other native breeds, it was observed that the Carora breed showed a lower value compared to the Reyna breed (7.2 years) from Nicaragua (54). This could be because some of the sires used for breeding may be advanced in age, or because their insemination doses have been stored and are still in use today, as is the case with the Brahman, Gyr, Guzerat, Indubrasil, Nelore, Sindi, and Tabapua, where GI values were greater in the sire-son pathway (6.80 and 13.79 years) and in the dam-son pathway (7.10 and 10.95 years) due to the low use of young sires, despite the existence of genetic improvement programmes (49).

The dam-son and dam-daughter pathways showed values of 5.5 to 6 years, respectively, which were high values compared to other dairy breeds in Canada (Holstein Frie-sian, Ayrshire, Jersey, and Brown Swiss) that showed values between 3 and 4 years (55), while when compared to beef breeds, the values were similar, such as in the Simmental breed in Brazil, whose maternal pathway showed values of 5.01–6.7 years (56). This difference could be due to the fact that in specialised dairy breeds, herd management was intensive with greater use of reproductive biotechnologies such as AI and embryo transfer (55, 57, 58). In addition, genomic evaluation is less common compared to specialised beef breeds, where management is more extensive (56, 59). Similarly, among the Zebu breeds in Brazil, only the Brahman breed showed a similar value of 5.20 years, while in the Gyr, Guzerat, Indu-brasil, Nelore, Sindi, and Tabapua breeds, the values were 7.03, 6.86, 7.96, 6.73, 6.87, and 6.65 years, respectively (49). In general, from a conservation point of view, a great GI value in the Carora breed could benefit the population, as it prevents an accelerated increase in inbreeding.

In general, the coefficient of inbreeding in the last period (2015–2024) showed an increasing trend, reaching values of 2.35 and 5.88% for the classical and recursive methods, respectively. Analysing the classic *F* value for Brazilian Zebu breeds born between 2005 and 2012, the values were similar (1.90–3.01%) except for the Indubrasil breed, with an F of 6.24% (49). In addition, in native breeds of Spain, the values were greater, such as the Negra Andaluza (7.23%), Vianesa (3.1%), Caldela (4.0%), Limia (4.10%), Frieiresa (3.7%), Berrenda negra (7.0%), and Berrenda Colorada (5.70%) (39, 40, 60). This difference in values could be due to the difference in the structure and management of these populations. For example, among the Zebu breeds of Brazil, the Gyr and Guzerat dairy breeds showed values between 2.04 and 2.39%, similar to those of the Carora breed in the present study. Meanwhile, in Ibero-American Creole breeds, such as Blanco Orejinegro, F was 1.32% (48), Romosinuano with *F* = 2.53% (42), and in the Criollo Limonero breed, *F* = 2.05% (43).

In the present study, the estimated increase in inbreeding in the last period was 0.43%, with an upward trend, which was a lower value compared to the Criollo Limonero breed of Venezuela (ΔF = 0.38%) (43), although similar to the Romosinuano breed of Colombia (ΔF = 0.49%); however, the value was low compared to the native Berrenda Negra and Colorado breeds (ΔF = 3.01% and ΔF = 4.02%, respectively) (40), Negra Andaluza (ΔF = 3.17) (39), and in native Italian breeds, such as the Calvana breed (ΔF = 2.54%), Mucca Pisana (ΔF = 2.70%), Pontremolese (ΔF = 3.42%), Sarda (ΔF = 3.00%), Sardo Bruna (ΔF = 2.64%), and Sardo Modicana (ΔF = 1.26%) breeds (61). This difference between Creole breeds from Latin America and native European breeds could be due to population size, pedigree integrity, and different breeding practices (Creole breeds have less integrity than native breeds). Despite this, the FAO (62) recommended that within small and at-risk populations, the increase in inbreeding per generation should be a maximum of 1% in order to maintain a Ne of 50 animals. Furthermore, if this value is higher, breeders should use more breeding animals alongside financial incentives, education, and the establishment of alternative parents, among other FAO recommendations.

Regarding the AR value (which reflects the average degree of genetic relationship between in-dividuals in a population) the Carora cattle showed a value of 4.04%, which was slightly greater than the Criollo Limonero breed, showing a value of AR = 3.10% (43) and other native Italian breeds such as the Sardinian (Sarda, Sardo Bruna and Sardo Modicana) showing values between 0.04 and 0.37% (61) and the Negra Andaluza breed from Spain showing a value of 2.40% (39), although this value was relatively low when compared to the native Italian breeds of Tuscan (Calvana, Mucca Pisana, and Pontremolese), which showed values between 6.39 and 10.54% (61). The AR values obtained in the Carora breed showed that inbreeding could increase dramatically in the near future if the average degree of genetic relationship between individuals continues to increase in the population.

The value of *α* in all periods showed negative values except in the last period, which was positive (α = 0.0034). This could be due to breeders having carried out ineffective matings, where individuals with greater than average inbreeding or coancestry mated (under random mating) more frequently than expected, and in the case that strategies to reduce inbreeding were applied, they were not effective enough. This effect was similar to that observed in other breeds, such as the Jersey breed in the United States (positive value) (63), the native Negra Andaluza (0.061) breed in Spain (39), and the Venezuelan Criollo Limonero cattle (0.0051), where α values were positive in two chronological periods (43).

The genetic conservation index (GCI) has been used in genetic improvement and conservation programmes to assess genetic diversity and long-term conservation potential, as it helps to identify individuals that contribute most to maintaining genetic variation within a population (64). A higher GCI value indicates a greater contribution to genetic diversity and, therefore, a better suitability for conservation efforts. The GCI value in the last period was 2.27, which was low compared to exotic breeds reared in Ecuador, such as the Charolais, which showed GCI values of 7.18 (52, 53), and Montbeliarde cattle (GCI = 3.12) (50), which were characterised by having a relatively small population. Furthermore, when compared to the Criollo Limonero breed from Venezuela (43), the GCI value was 4.5 times greater than Carora, despite the fact that the population size in Criollo Limonero was 462 while Carora had 21,350 individuals, which could indicate better management by cattle breeders. Nonetheless, the value obtained in the Carora breed was greater than other specialised breeds, such as the Holstein Friesian in Ecuador, which showed a value of 1.42 (51).

During the last period (2015–2024), the Ne value showed a reduction in the three methods analysed, suggesting a genetic diversity loss and a detriment in the reproductive performance (as shown in the results of the present study). The Ne values in this last period were between 100 and 150 individuals, a value that can be considered normal according to the FAO recommendations (62, 65), which established a minimum Ne of 50 individuals to maintain a maximum increase in F of 1% per generation. Furthermore, with the advent of modern analysis techniques, such as genomics (66), it was observed that a minimum of 50 individuals within local populations or in conservation programmes is necessary to estimate Ne, always taking into account the particular factors of the populations, such as substructures and demographic history, among others. In the Carora breed, it is crucial to implement conservation strategies to reverse this trend and ensure the long-term viability of the population.

Regarding the effective number of equivalent founders, this value was reduced to 58.69 in the last period, which is low compared to other native breeds in Spain, such as the Negra Andaluza (fe = 91.14) and Berrenda Colorada (fe = 140) (39, 40). This fact shows a greater risk of genetic diversity loss in the Carora population due to the greater influence of a few individuals on the gene pool, which may increase inbreeding. In addition, this population could be experiencing less diversified reproductive management or more severe historical bottleneck events. To mitigate this phenomenon, it is necessary to implement selection strategies that prioritise the inclusion of less-represented genetic lines of sires or dams and avoid the overuse of breeding stock, especially through artificial insemination. In addition, collaborative conservation programmes with other populations could help restore any diversity losses and reduce risks. Similarly, the effective number of ancestors (fa) remained approximately 28 in all periods, indicating a strong dependence on a small group of key ancestors in the population and confirming a further narrowing of the gene pool with respect to the number of founders (fe).

The fa/fe ratio value has remained approximately 0.48, indicating that this breed was experiencing a bottleneck similar to the Negra Andaluza (fa/fe = 0.46) and Criollo Limonero (fa/fe = 0.49) breeds; however, the value was lower than in native beef breeds in Spain, Berrenda Negra and Colorada (40), Tabapua (67), Lageana (68), and Criollo Tropical (69) in Mexico, with values greater than 1. In the Carora breed, a possible bottleneck could be the low identification and availability of genetic lines within the breed that were distributed through the insemination centre, added to the use of older breeding animals, as demonstrated by the generational interval value (Figure 2). In the last period, the fg value was 27.74. Considering that this parameter indicates the maintenance of founder genes in the population for a particular locus, it could reveal the causes of gene loss generated by segregation (70). When comparing the fg value in the Carora breed with that of the Negra Andaluza breed (46.83) (39), it is a lower value, indicating a greater foundational genetic diversity loss due to the unbalanced use of breeding stock, historical or recent bottlenecks, and/or a lack of active conservation strategies. These effects lead to the need to balance the contributions of breeding stock, generate controlled introgression, and apply genomic technology. The fa, fe, and fg values were consistent with the low number of ancestors that explain 25, 50, and 75% of the current genetic diversity (3, 9, and 32, respectively), confirming the concentration of inheritance in a few individuals.

Finally, the genetic diversity loss (1-GD) showed an increasing trend, varying between 0.73 and 1.80%, while the loss due to the unequal contribution of founders was close to half of these values among the evaluated periods, when compared to the values of the Berrenda Negra and Colorada breed in Spain (40), showing a value of 0.4 to 0.1%, respectively, and the Romosinuano breed in Mexico with estimated values of 2.5–3.7% (6), which were considered high; however, this effect was not as pronounced as in the Criollo Limonero breed in Venezuela (4.31%) (43). This fact determines that the primary cause of genetic diversity loss could be genetic drift and bottlenecking. Therefore, there is a need to develop more effective conservation strategies to mitigate these effects. Finally, the GD loss due to the unequal contribution of the founders (0.85%) in the last period was high compared to the Berrenda Negra and Colorada breeds, which showed values of 0.5 and 0.2%, respectively.

## 5 Conclusion

The Carora cattle breed is undergoing a process of genetic diversity loss due to genetic drift and bottlenecks, which creates the need to implement short- and medium-term strategies to prevent this fact. The positive value of *α* indicates that livestock breeders are mating animals with a higher than average kinship, which has led to an increase in inbreeding and a low average GCI value, making it imperative to implement a targeted mating strategy considering the lowest possible kinship. Finally, it is recommended to monitor mating to maintain genetic diversity and avoid a further increase in inbreeding values.

## Data Availability

The original contributions presented in the study are included in the article/supplementary material, and further inquiries can be directed to the corresponding authors.
